# The current landscape of CAR T-cell therapy for solid tumors: Mechanisms, research progress, challenges, and counterstrategies

**DOI:** 10.3389/fimmu.2023.1113882

**Published:** 2023-03-20

**Authors:** Amin Daei Sorkhabi, Leila Mohamed Khosroshahi, Aila Sarkesh, Amirhossein Mardi, Ali Aghebati-Maleki, Leili Aghebati-Maleki, Behzad Baradaran

**Affiliations:** ^1^ Student Research Committee, Tabriz University of Medical Sciences, Tabriz, Iran; ^2^ Immunology Research Center, Tabriz University of Medical Sciences, Tabriz, Iran; ^3^ Department of Immunology, School of Medicine, Tehran University of Medical Sciences, Tehran, Iran; ^4^ Stem Cell Research Center, Tabriz University of Medical Science, Tabriz, Iran; ^5^ Department of Immunology, School of Medicine, Tabriz University of Medical Sciences, Tabriz, Iran

**Keywords:** chimeric antigen receptor, CAR T-cell, immunotherapy, solid tumors, challenges, clinical trials

## Abstract

The successful outcomes of chimeric antigen receptor (CAR) T-cell therapy in treating hematologic cancers have increased the previously unprecedented excitement to use this innovative approach in treating various forms of human cancers. Although researchers have put a lot of work into maximizing the effectiveness of these cells in the context of solid tumors, few studies have discussed challenges and potential strategies to overcome them. Restricted trafficking and infiltration into the tumor site, hypoxic and immunosuppressive tumor microenvironment (TME), antigen escape and heterogeneity, CAR T-cell exhaustion, and severe life-threatening toxicities are a few of the major obstacles facing CAR T-cells. CAR designs will need to go beyond the traditional architectures in order to get over these limitations and broaden their applicability to a larger range of malignancies. To enhance the safety, effectiveness, and applicability of this treatment modality, researchers are addressing the present challenges with a wide variety of engineering strategies as well as integrating several therapeutic tactics. In this study, we reviewed the antigens that CAR T-cells have been clinically trained to recognize, as well as counterstrategies to overcome the limitations of CAR T-cell therapy, such as recent advances in CAR T-cell engineering and the use of several therapies in combination to optimize their clinical efficacy in solid tumors.

## 1 Introduction

Through ages and decades, conventional therapies such as surgery, chemotherapy, and radiation and the newly emerged targeted therapies have formed the mainstay of cancer treatment. Despite their promising results, poor prognosis is intertwined with many malignancies, the complications of which have been addressed owing to targeted anticancer approaches that permit the therapies to become individualized with a higher rate of success (1, 2). Since an individual’s immune system is subjected to defeating his/her cancer, immunotherapy has become the definitive example of personalized medicine owing to a radical change in attitudes towards cancer treatment. The scope of immunotherapy has been widened adequately to cover not only monoclonal antibody therapy but also tumor vaccinations, immune checkpoint blockades, bispecific antibodies, tumor-infiltrating lymphocytes (TILs), and even chimeric antigen receptor (CAR) T-cell therapies. T-cells lie at the heart of the adaptive immune system for both regulating cytotoxic effects and retaining the everlasting cellular memory of particular antigens (3). Although the tumor-specific TILs are produced in patients’ cells, being enclosed in the TME renders them anergic and non-functional (4). Endogenously, the interplay between major histocompatibility complex (MHC)-displayed peptides and their T-cell receptor (TCR) triggers T-cells (5); nevertheless, the activation of CAR T-cells is contingent upon the presence of tumor-associated or tumor-specific antigens (TAA and TSA), respectively. The infusion of a targeting domain [single chain variable fragment (scFv)] to the signaling domain of a T-cell yields CAR T-cells, known as a living drug (6, 7). In fact, CAR is an elaborate amalgam of an antibody or ligand-derived targeting ectodomain and a hinge, a trans-membrane domain, as well as intracellular T-cell signaling domains. The peculiarities of the antigen, previously determined by the targeting domain, are transferred by CARs if they are in turn, expressed by a T-cell (1, 8). What makes CARs superior to TCRs (conventional T-cell receptors) is that, unlike TCRs, which require an MHC-dependent manner to identify antigens, any type of target either as a protein or non-protein being expressed on the cell surface can receive the effector function of a T-cell when CARs play the role. This advantageous feature obviates any need for antigen processing and presentation and thus, non-classical T-cell targets such as carbohydrates can be addressed as well (9). The curative traits of the CAR T-cell approach have earned universal acceptance and show promising potential for adoptive T-cell therapy owing to sidestepping human MHC restriction (10).

## 2 CAR T-cell therapy design

Efficient, firm, steady, and secure gene transfer platforms boost the success rate of CAR T-cell therapy. Having been isolated *via* leukapheresis, autologous T-cells get harvested and then undergo *ex vivo* genetic modification through viral and non-viral transfection approaches. Once the quality control testing is implemented on the expanded and prepared T-cells, the patient is set to receive lymphodepleting chemotherapy combined with CAR T-cell infusion. It was Eshhar’s team at the Weizmann Institute of Science in Israel who first devised a chimeric receptor (11). The antigen-binding moiety coupled with a spacer constitutes the extracellular domain of CARs (8). Such moieties can belong to three categories: a) an scFv originated from antibodies; b) a human Fab fragment, singled out from phage display libraries; or c) nature ligands that make use of their cognate receptor. Mouse monoclonal antibodies (mAbs), humanized Abs, or entirely human Abs are the sources of scFv, a variable mAb fragment with the ability to recognize and bind to TAAs expressed on the cell surface of tumors. Quite opposite to normal TCRs, not only unprocessed antigens but also carbohydrate glycolipid structures that are normally expressed on tumors’ cell surfaces can be identified by CARs even in the absence of antigen presentation *via* MHC (12). Redirected recognition of the target T-cell is achieved when CAR T-cells of both cluster of differentiation (CD)8^+^ and CD4^+^ subsets surmount MHC class I and II barriers. Predominantly, two passages are utilized for cytolysis to eliminate CAR-mediated tumors, namely perforin and granzyme exocytosis, and, on a small scale, death receptor signaling *via* Fas/Fas-ligand (Fas-L) or tumor necrosis factor (TNF)/TNF-receptor (TNF-R) (9). The space connecting the antigen binding domain and the transmembrane domain is called the spacer. The most straightforward form of the spacer is the hinge region of immunoglobulin (Ig)G1, which is quite apt for the majority of scFv-based constructs (13). The transmembrane domain, which is commonly derived from CD8 or CD28, links the extracellular antigen binding domain to the intracellular signaling domain, with the receptor CD28 transmembrane being particularly known for its high stability (14). CD3ζ is regarded as the most common component of the intracellular domain which sparks off the activation and function of the T-cell by emitting its first signal (15). The heightened production of cytokines like interleukin (IL)-2 as well as the *in vivo* proliferation and durability of the T-cell is initiated as soon as the second signals, co-stimulatory signals, are produced. The functional behavior of CARs is immensely influenced by the intracellular signaling domain, which is the reason behind its thorough preclinical and clinical assessment (16).

Since 1989, the structure of the intracellular domain has been the determining factor of four generations that CAR T-cells can be allotted to (17). ζ chain of complex TCR/CD3 (CD3ζ) is the distinguishing aspect of the first generation. However, the dual signal for stimulating the T-cell is what differentiates the second generation in which either antigen recognition or co-stimulatory molecule ignites the activation (18). For instance, CD28/B7, a co-stimulatory molecule, can elevate IL-2 synthesis to turn on T-cells and deter apoptosis (13). Intensified responses are the trademark of the third generation of CARs, arising from the merge of the sequences of co-stimulatory signals like OX40 (CD134), CD28, 4-1BB (CD137), CD27, DNAX-activating protein 10 (DAP10) with CD3ζ (19, 20). The amalgam of multiple co-stimulatory signals reinforces the function of CAR T-cells thanks to the promoted cytokine production, T-cells proliferation and eradication as long as antigen exposure exhibits a recurring pattern (16). Despite this, the relative enhancement in the patients’ conditions has not been proved as compared to the second generation of CARs, which necessitates complementary investigations to affirm the safety and efficacy of the third generation (8). It is suggested that streamlined CARs, CAR T-cells, can be deployed for universal cytokine-initiated killing (TRUCK). Transient products may originate from TRUCK cells, namely IL-12 or interferon-gamma (IFN-γ) (21). Although the former can provoke innate immune responses while remaining undetectable to CAR T-cells, antigen-dependent eradication of tumor cells can be set off by IFN-γ and interferon-gamma receptor (IFN-γR) in particular, with its expression being manifested in tumor stroma (21, 22). The fourth generation of CARs is fundamentally identical to the second-generation CARs, but it possesses a protein, such as (IL-12) that is constitutively or inducibly expressed once CAR activation initiates. T-cells redirected for the TRUCKs are considered when the fourth-generation CARs are in charge of transducing T-cells. The arousal of these CARs fosters the production and secretion of the favorable cytokine to further advance tumor killing through a variety of synergistic mechanisms, such as exocytosis (perforin, granzyme) or death ligand–death receptor (Fas–FasL, TNF-related apoptosis-inducing ligand (TRAIL)) systems (6, 23). A fifth generation of CARs is presently under scrutiny; they have the second generation of CARs as their basis; however, in order for the transcription factor signal transducers and activators of transcription 3 (STAT3), they encompass a truncated cytoplasmic IL-2 receptor β-chain domain with a binding site. The antigen-specific activation of this receptor concomitantly ignites TCR (through the CD3ζ domains), co-stimulatory (CD28 domain) and cytokine (Janus kinase (JAK)–STAT3/5) signaling, culminating in the effective provision of all three synergistic signals needed physiologically to stir a thorough T-cell stimulation and multiplication (24–26).

Synergistic and reinforced killing power are the upshots of the application of biphasic CAR (tandem CAR, TanCAR) by which only one transgenic receptor identifies two distinct antigens whose recognition domains are located in tandem with a flexible hinge lying in between. As a direct consequence, antigen loss and tumor escaping are avoided since the downregulation and mutation of one target antigen does not inactivate TanCAR and thus, the cytolytic capacity of the T-cells is maintained (27). In an attempt to augment anti-tumor effects, researchers suggested dual specific CARs, in which a homogenous T-cell population co-expresses two distinct CARs, with one detecting a distinct antigen and the other supplying complementary signals. Such a method paves the way for tumor barcoding, which will only eradicate double-antigen positive tumors. In other words, suboptimal CD3ζ-mediated activation is produced in a CAR once an antigen and a chimeric co-stimulatory receptor encompassing only CD28 and 4-1BB bind with each other and in turn, make CAR T-cells to detect a second antigen, all of which giving rise to CAR T-cell specificity and averting off-target effects, and T-cells set in motion upon encountering CARs’ targets (28, 29).

Aside from antigen-specific methods, two universal CAR systems have also been introduced, which entail CARs with ScFv for avidin (30) or anti-fluorescein isothiocyanate (FITC) (23) that can detect all tumors integrated with biotinylated or bound by FITC-labeled antitumor Abs. In order to leave off-target tissues intact, an antigen-specific inhibitory CAR (iCAR) limits the function of T-cells to tumor tissues specifically. Inadequate T-cells specificity can be prevented by the dynamic, self-regulating safety switch of iCARs. Such T-cells comprise not only a tumor-antigen targeting CAR, but also an iCAR that attacks an off-target tissue antigen in combination with an intracellular strong acute inhibitory signaling domain based on the programmed death-1 (PD-1) or cytotoxic T lymphocyte-associated antigen 4 (CTLA-4) molecules. Through their interaction with the off-target tissue antigen, these cells have the capacity to discriminate and inhibit cytokine release, cytotoxicity, and proliferation (31, 32). In order to transcend limitations (e.g., controllability, flexibility, specificity) in mainstream CAR T-cell therapies, novel receptor designs have been devised. For instance, CAR activities are regulated by drug-inducible on-and-kill switches (33). Likewise, flexibility in antigen recognition is heightened by dissociating the antigen-recognition motif of CARs from the signaling motif; hence, a universal receptor becomes the shared ground for all interactions, extending the range of targeted antigens without any need to re-engineer the immune cells (23, 34, 35). Finally, combinatorial antigen sensing, which targets two tumor-specific antigens (36, 37) and lowers antigen escape rate contributes to enhancing tumor specificity (27, 38). CAR T-cell therapy can gain boosted safety and efficacy provided that these features are exploited, but unfortunately, such advanced CARs fail to accommodate all qualities in a single system; what is more, the diversity of immune responses can be restricted due to the fixed number of activated signaling pathways and cell types. To address these shortcomings, a split, universal, and programmable (SUPRA) CAR system with a universal receptor that can be expressed on T-cells and a tumor-targeting scFv adaptor molecule is developed.

## 3 The administrating procedure of CAR T-cells

The first stage in the collection and administration of CAR T-cells is the identification of ideal possibilities for commercial CAR T-cell products or clinical tests. A variety of factors influencing the outcomes of CAR T-cell therapy must be considered, including early referral to centers, tumor burden, previous therapies, and performance status. If clinical trials are not welcomed by volunteers, financial coordinators conduct some evaluations on patients to be submitted to insurance companies or hospital financial clearance. Subsequently, mononuclear cell collection is conducted *via* apheresis. An absolute lymphocyte count of 100–200/mL in 14 days after the final salvage regimen is the prerequisite for carrying out clinical trials with approved commercial products (39, 40). Following the apheresis, the manufacturing factories receive cryopreserved and fresh cells to conduct further processes such as T-cell development, genetic manipulation through the retroviral or lentiviral transduction, quality control trial, and cryopreservation of the ultimately developed T-cell product. In the course of this 2-4 week period, the malignancy of patients undergo bridging therapies prior to lymphodepleting chemotherapy to optimize the immune context for the multiplication of infused CAR T-cells, and ultimately, CAR T-cell-associated toxicities are scanned (41).

## 4 Target antigens for CAR T-cell therapy of solid tumors in clinical trials

### 4.1 EGFRvIII

The development of CARs has attracted a great deal of interest and attention owing to the favorable results yielded by the application of CD19 CARs in treating tumors. A large number of tumor-related antigens are studied to raise the likelihood of optimal efficacy. The pathogenesis of malignant glioblastoma (GB) is attributed to the over-expression of epidermal growth factor receptor (EGFR) and EGFR variant III (EGFRvIII) in a host of cancer types (42, 43). Normally, survival, invasion, angiogenesis, and resistance against radiation and chemotherapy emanate from the expression of EGFRvIII in a cell (44). Having produced noticeable anti-tumor outcomes in pre-clinical experiments, EGFRvIII-specific CAR T-cells are being assessed for clinical trials (45). By exerting some modifications, EGFR can still possess a cetuximab binding site but may lose its domains I and II as well as its cytoplasmic tail, thus its cetuximab can identify the truncated EGFR (huEGFRt), and consequently, the CAR T-cells expressing the truncated EGFR can be singled out, traced, and ablated following the administration of cetuximab (46). EGFRvIII is expressed in 25-30% of newly diagnosed GB tumors and is being investigated in clinical trials as a target for GB tumor treatment (47). Despite feasible cell manufacturing and clinical safety of intravenous administration, two clinical trials on GB patients investigating EGFRvIII-targeting CAR T-cells co-stimulated with 4-1BB alone (NCT02209376) (48) or in conjunction with CD28 (NCT01454596) (49) failed to demonstrate any radiographic responses. Likewise, in the phase II clinical trial administering EGFR-CAR T-cell therapy in patients with EGFR-positive relapsed/refractory non-small lung cancer (NSCLC), two patients achieved partial remission and five patients had stable disease for two to eight months with no substantial adverse effect (NCT01869166). Yet, clinical studies are underway to investigate the therapeutic efficacy of C-X-C chemokine receptor type 5 (CXCR5)-expressing EGFR-CAR T-cells, transforming growth factor-beta (TGF-β) knockout EGFR-CAR T-cells, and IL-12-secreting EGFR-CAR T-cells in NSCLC, advanced biliary tract cancer, and colorectal cancer (CRC), respectively (NCT04153799, NCT04976218, and NCT03542799).

### 4.2 IL13Rα2

The diminished survival rate of patients is also linked to a glioma-associated antigen named Interleukin 13 receptor α2 (IL13Rα2). In a study, treatment with CAR T-cell brought about the regression of tumors along with a corresponding rise in cytokines and immune cells (50). Since IL13Rα2-specific CARs can also detect IL13Rα1, IL13Rα1-specific scFv is designated as an antigen binding domain, leading to augmented specificity (51). Positron emission tomography (PET) imaging studies have shown that IL13Rα2-specific CAR T-cells can effectively traffic to the brain parenchyma, specifically tumor regions (52). Clinical trials of IL13Rα2-CAR T-cell therapy in GB patients were basically initiated by local delivery of first-generation anti-IL13Rα2 CAR T-cells into the resection cavity of GBs and were improved with a second-generation, 4-1-BB co-stimulated construct, demonstrating promises for GB treatment and fair tolerance (NCT00730613) (53). According to another research, although the intracranial and spinal tumor regression was remarkable in clinical and imaging findings, the complete response was temporary, lasting only around 7.5 months, and the tumor recurred in new regions (NCT02208362) (50).

### 4.3 Mesothelin

Although the precise mechanisms have yet to be fully understood, current evidence suggests potential implications for mesothelin in tumor cell adhesion, progression, proliferation, survival, and resistance to chemotherapy (54). Mesothelin overexpression has been documented in a variety of solid tumors, and CAR T-cells targeting mesothelin have been clinically investigated in mesothelioma, epithelial ovarian cancer, pancreatic ductal adenocarcinoma (PDAC), lung cancers, uterine cancers, triple-negative breast cancer (TNBC), gastric cancer (GC), CRC, esophagus cancer, hepatocellular carcinoma (HCC), as well as neuroendocrine tumors/Merkel cell carcinoma. Given the strong preclinical evidence suggestive of the robust anti-tumor activity of anti-mesothelin CAR T-cells, their poor persistence in the TME has hampered their broad clinical applicability in mesothelin-overexpressing tumors; however, research into compensatory measures for these challenges is currently underway, as will be discussed in the following sections.

Multiple phase I and II clinical trials have been undertaken to investigate the monotherapy with anti-mesothelin CAR T-cells or its combination with other therapies including immune checkpoint inhibitors (ICIs) and standard treatments. In this way, the first phase I clinical trial evaluating CAR T-cells electroporated with mesothelin mRNA demonstrated their effective anti-tumor activity in three patients with malignant pleural mesothelioma (MPM) and one patient with PDAC, while inducing no serious adverse complications, with the exception for incidence of severe anaphylactic reaction in one of the MPM patients (NCT01355965) (55). Similarly, in another trial of six PDAC patients, intravenous delivery of anti-mesothelin CAR T-cells resulted in stable disease, with progression-free survival (PFS) times of 3.8 and 5.4 months in two patients, respectively, and no adverse complications associated with off-tumor toxicities. Further PET imaging studies in these patients indicated that post-treatment metabolic active volume was stable in three patients and declined by 69.2% in one patient with biopsy-proven mesothelin expression (56). In addition, 15 patients with MPM, PDAC, or ovarian cancer were given lentiviral-transduced anti-mesothelin (murine SS1 scFv) CD3ζ/4-1BB CAR T-cells with or without cyclophosphamide. It was well-tolerated and led to stable disease in 11 patients; nevertheless, CAR T-cells expansion in the blood peaked on days 6-14, and in patients pre-treated with cyclophosphamide, it persisted for only 28 days (NCT02159716) (57). Given the short-term anti-tumor effects of anti-mesothelin CAR T-cells demonstrated in these trials, researchers have considered combining CAR T-cell immunotherapy with ICIs. In this context, intrapleural delivery of anti-mesothelin CAR T-cells followed by pembrolizumab (anti-PD-1 mAb) to 18 MPM patients resulted in eight patients having stable disease for more than six months and two patients having complete metabolic response on PET scan, with CAR T-cells detectable in peripheral blood for more than 100 days in 39% of patients (NCT02414269) (58). Furthermore, in another phase I clinical trial, researchers applied CRISPR-Cas9 technology to gene modify lentiviral-transduced anti-mesothelin CAR T-cells to disrupt PD-1 expression and infused to 15 patients with metastatic mesothelin-positive solid tumors. They discovered the best overall response of stable disease in two patients and no indications of autoimmune reaction, on-target/off-target toxicities, or unexpected toxicities after infusion, but CAR T-cells did not persist more than six weeks in peripheral blood, at the tumor site, or in effusion samples of almost all patients after the initial infusion (NCT03545815) (59).

### 4.4 HER2

According to studies, human epidermal growth factor receptor 2 (HER2) is overexpressed in breast, gastric, ductal, pancreatic, NSCLC, and GB tumors and has been linked to carcinogenesis, suggesting that it might be exploited as a prognostic marker and a therapeutic target in cancer treatment (60).

Although CAR T-cells directed toward the HER2-expressing tumors have been extensively studied in clinical trials, safety concerns have emerged following the death of a CRC patient who received 1×10^10^ third-generation HER2-CAR T-cells (61). On the other hand, the phase 1 open-label dose-escalation trial on 17 patients with advanced HER2-positive GB discovered that 1 or more infusions of 1×10^8^ second-generation HER2-CAR cytomegalovirus (CMV) pp65-bispecific cytotoxic T lymphocytes (CTLs) were well tolerated without treatment-related severe toxicities. Of 16 evaluable patients, 1 had a partial response for more than nine months, seven had stable disease lasting eight weeks to 29 months, and the condition of eight progressed after therapy. Despite this, CAR T-cell levels in the blood dropped month by month, and two patients remained positive after 12 months, with neither patient being positive after 18 months, demonstrating that HER2-CAR T-cells did not expand upon delivery but survived for almost a year (NCT01109095) (62, 63). Furthermore, the phase I clinical trial assessing the efficacy and safety of locoregional delivery of HER2-CAR T-cells *via* central nervous system (CNS) catheter to the tumor cavity or the ventricular system of children and young adults with R/R CNS tumors found significant immune responses as demonstrated by increment in C-C motif chemokine ligand 2 (CCL2) and C-X-C motif chemokine ligand 10 (CXCL10) levels, with no dose-limiting adverse reactions (NCT03500991) (64). As well, in phase I clinical study, 11 patients with advanced pancreatic and biliary tract cancers who had received cyclophosphamide and nanoparticle albumin-bound paclitaxel (nab-paclitaxel) preconditioning were given 1 to 2 cycles of 2.1 10^6^/kg HER2-CAR T-cells. Preliminary findings reported one case of grade-3 febrile syndrome during infusion and another for upper gastrointestinal bleeding, with clinical efficacy of partial response after 4.5 months and five patients achieving stable disease (NCT01935843) (65).

### 4.5 PSMA

Since prostate-specific membrane antigen (PSMA) is secreted from the majority of prostate cancer (PCa) cells and tumor-associated neo-vasculatures, antiangiogenic and anti-tumor effects can presumably deal with PSMA-CARs (NCT00664196) (66). Targeting the PSMA expressed by non-cancerous tissues may spark negative signaling to the PSMA-specific dual target CAR T-cells with the co-stimulatory molecules of PD-1 or CTLA-4 (66, 67) and thus, enhancing the specificity of CAR T-cells. This so-called strategy of iCARs incorporates the safety factor to improve antigen recognition (68).

In a phase I clinical trial, PSMA-specific CAR T-cells were administered to five PCa patients. Two of the patients exhibited prostate-specific antigen responses, and the patients’ clinical outcomes had a negative correlation with the level of CAR T-cell infusion and a positive correlation with IL-2. Thus, the phase II clinical study was planned to combine PSMA-specific CAR T-cells with moderate doses of IL-2 supplementation, which were found to be essential for CAR T-cell anti-tumor activities in the TME (69). Further studies are being carried out to investigate the therapeutic efficacy and safety of PSMA-CAR T-cells in PCa treatment (NCT04053062, NCT04227275, NCT04249947, NCT04429451). Nonetheless, clinical trials are not confined to PCa; PSMA-specific CAR T-cells are also being studied for the treatment of advanced or metastatic urothelial bladder cancer (NCT03185468) and relapsed and refractory neuroblastoma (NCT04637503).

### 4.6 Mucin-1

Cell membrane mucin-1 (MUC1), a product of aberrant glycoform expression, is one of the big-sized proteins capable of transferring O-glycan proteins that are over-expressed by a large number of adenocarcinomas (70, 71). mAb (5E5)-based CARs can select MUC1 glycopeptide epitope as a target and potentially kill pancreatic tumors (NCT02587689) (72, 73). CAR T-cells gain further vigor thanks to pathophysiologic and therapeutic links of IL-4 to cancers. Intensified resistance against immunosuppressive cytokines as well as heightened anti-tumor efficacy is the manifested traits of MUC1-CAR T-cells engineered with IL-4 receptor ectodomain (74, 75).

A phase I clinical trial employing two distinct designs of MUC1-CAR T-cells derived from the S MUC1 antibody (SM3) antibody evidenced serum cytokine responses and no adverse effects in a patient with metastatic seminal vesicle cancer (76). Similarly, PD-1 deficient CAR T-cells with the same specificity (SM3 scFv) were tested in the treatment of NSCLC and found to be well-tolerated and safe (NCT03525782) (77). In addition, a multi-center first-in-human phase I clinical trial investigated the safety and efficacy of TnMUC1-CAR T-cells with CD2 costimulatory domain in the treatment of 6 patients with TnMUC positive solid tumors including metastatic treatment-resistant ovarian cancer, pancreatic adenocarcinoma, TNBC, or NSCLC. They found that CAR T-cells were expanded in all patients, particularly those who had undergone lymphodepleting chemotherapy with fludarabine and cyclophosphamide, and that the intervention was safe, with no on-target/off-tumor toxicity (NCT04025216) (78). The efficacy of the treatment is scheduled to be evaluated in a second expansion phase that encompasses 72 more patients.

### 4.7 GD2

Ganglioside GD2 is a tumor-associated carbohydrate surface antigen that, unlike other gangliosides that are expressed by most normal tissues, is preferentially overexpressed by the vast majority of neuroblastomas, melanomas, retinoblastomas, and Ewing sarcomas (79). GD2 not only enhances tumorigenesis by inducing cellular proliferation, migration, and apoptosis resistance, but it also exhibits immunosuppressive properties that hinder T-cell activation and dendritic cell maturation upon its release into circulation (80).

For the first time, GD2 targeting Epstein-Barr virus (EBV)-specific CAR T-cells were delivered to eight patients with neuroblastoma, leading to tumor necrosis or regression in four patients with no on-target/off-target toxicity (81). Further long-term follow-up analysis by the same researchers suggested that CAR T-cells in the intervention group persisted for at least four years (NCT00085930) (82). The fourth-generation GD2-CAR T-cells with CD28/4-1BB/CD3ζ-iCasp9 signaling domains were also investigated in the phase I clinical trial on 10 pediatric patients with refractory and/or recurrent neuroblastoma. The therapy resulted in a 25-month median overall survival (OS) time and an 8-month median PFS time with minimal to no toxicities (NCT02765243) (83). Another study on eight patients with osteosarcoma and three with neuroblastoma discovered that clinical response in patients treated with GD2-CAR T-cells is negatively associated with myeloid-derived suppressor cells (MDSCs) level in their peripheral blood mononuclear cells (PBMCs), highlighting the MDSCs as a target for the development of combination therapies (84).

Furthermore, in phase I clinical trial, GD2-specific CAR T-cells were examined for the treatment of patients with H3K27M-mutated diffuse midline gliomas (DMG), and it was discovered that a single dose intravenous administration of 1×10^6^/kg GD2-specific CAR T-cells can significantly enhance the release of proinflammatory cytokines and chemokines such as CXCL9, CCL2, TNF-α, and IFN-γ in the TME, resulting in radiographic and clinical improvement in three out of four patients (85). After the first intravenous treatment, the tumor volume in one of the treated patients with spinal cord DMG was diminished by 90%, and after the second intracerebroventricular infusion, the tumor dimensions were reduced by 80% (85). Additionally, no on-target or off-target toxicity was observed, and the researchers discovered that neurocritical care precautions and multimodal therapy could be applied to safely address predicted tumor inflammation-associated neurotoxicity (TIAN) (NCT04196413) (85).

### 4.8 NKG2D

The natural killer group 2D (NKG2D) receptor is a crucial regulator of effector immune cell function, activating robust cytotoxic pathways against cells expressing its stress-induced ligands even in the presence of normal concentrations of inhibitory MHC-I molecules. It is an intriguing target for the clinical research of novel therapeutics including NKG2D-CAR T-cells owing to its functional implications in innate and adaptive immunity against stressed cells (both infected and malignant cells) and the overexpression of its ligands on tumor cells (86). NKG2D was shown to be a widely overexpressed target in CRC based on data from preclinical models. CYAD-101, a CAR T receptor encoding NKG2D receptor, is being investigated in a phase 1 trial for safety and tolerability. In this study, two patients showed a partial response and nine had stable disease after receiving three doses of CYAD-101 cells following conventional treatment (NCT03692429) (87).

### 4.9 CLDN18.2

Claudin 18.2 (CLDN18.2) is the gastric isoform of CLDN18, a tight junction protein. It is extensively expressed in a variety of malignancies, particularly those of the digestive system, providing it a possible anti-tumor therapeutic target (88).

Preclinical research has been carried out to determine whether CAR T-cells redirected against the CLDN18.2 have the capacity to be employed as therapeutic agents in cancer therapy. To this end, CLDN18.2-CAR T-cells were created employing the scFvs as targeting moieties after the development of the CLDN18.2-specific scFv-containing humanized antibodies hu8E5 and hu8E5-2I. CLDN18.2-positive GC patient-derived xenograft (PDX) mice was treated with hu8E5-2I-28Z-CAR T-cells that persisted *in vivo* and effectively invaded tumor tissues, culminating in partial or total tumor eradication (89). Consequently, CLDN18.2-CAR T-cells may be a viable therapeutic option for GC and other CLDN18.2-positive malignancies. So far, several clinical trials in different phases evaluating CLDN18.2-CAR T-cell therapy have been launched.

According to preliminary findings from an investigator-initiated study, CT041 (a potential anti-CLDN18.2-targeted autologous CAR T-cell product) exhibited an appropriate safety profile and promising anticancer activity (CT041-CG4003 NCT03159819, NCT03874897). Also, the interim results of an ongoing, phase 1 clinical trial of CT041 in patients with previously treated, CLDN18.2-positive digestive system malignancies revealed that CT041 offers promising efficacy with an acceptable safety profile. The primary goal of this trial was to investigate safety following CT041 infusion; secondary goals included CT041 efficacy, pharmacokinetics, and immunogenicity. Thirty-seven patients were treated with one of three CT041 doses: 2.5×10^8^, 3.75×10^8^, or 5.0×10^8^ cells. Hematologic toxicity of grade-3 or above was observed in all patients, and grade-1 or 2 cytokine release syndromes (CRS) occurred in 94.6% of patients. There were no reports of grade 3 or higher CRS or neurotoxicity, treatment-related deaths or dose-limiting toxicities (DLTs). The objective response rate (ORR) and disease control rate (DCR) in GC patients were 57.1% and 75.0%, respectively, with a 6-month OS rate of 81.2%. According to the findings of this trial, CT041 showed potential efficacy and an acceptable safety profile in patients with CLDN18.2-positive digestive system malignancies, particularly in those with GC (NCT03874897) (90). Further studies are being carried out to evaluate the safety and potential therapeutic efficacy of CLDN18.2-redirected CAR T-cells in the treatment of GC, pancreatic cancer, and gastroesophageal junction adenocarcinoma (NCT05583201, NCT05472857, NCT04404595, NCT04581473, etc.).

### 4.10 CEA

Carcinoembryonic antigen (CEA) is a glycosylphosphatidylinositol-anchored glycoprotein that is overexpressed in more than 80% of CRC patients. Furthermore, it is abundantly expressed on the cell surfaces of numerous human cancers of epithelial origin, including ovaries, pancreas, stomach, and lung carcinomas (91). A study investigating CEA-targeted CAR T-cells for metastatic CRC and other CEA-positive malignancies is now underway. In this study, no serious adverse effects of CAR T-cell therapy have been documented, although they persisted in circulation for only a few days to a few weeks, so all participants had undetectable levels of CEA-targeted CAR T-cells 4-6 weeks post-infusion (NCT03682744).

Since CEA is found on diverse epithelial cells in multiple organs, researchers believe that it is critical to address the possibility of on-target off-tumor toxicity. To ensure efficient delivery and reduce the risk of severe toxicity, several studies are looking into administering CAR T-cells directly into the hepatic artery. Intrahepatic administration of anti-CEA CAR T-cells was tested in individuals with CEA-positive liver metastases in the phase I study (NCT01373047). There were no grade 3 or 4 adverse events associated with hepatic artery infusions (HAIs) of CAR T-cell, and one patient was still living with stable disease after 23 months, whereas five patients died of progressing disease. In four of six patients, biopsies revealed an increase in liver metastases, necrosis, or fibrosis (NCT01373047) (92). The following phase 1b trial demonstrated that severe neurotoxicity or CRS were not associated with the HAI delivery of CAR T-cells, and the average survival time was 8 months. While serum CEA levels were either steady or declined in all patients, liver metastases after HAI delivery of CAR T-cells displayed reduced levels of indoleamine 2,3-dioxygenase (IDO), granulocyte-macrophage colony-stimulating factor receptor (GM-CSF-R), and PD-L1 (NCT02416466) (93).

According to a patient case study, anti-CEA CAR T-cells were delivered into the hepatic artery using pressure-enabled drug delivery (PEDD) technology with no severe or off-target adverse effects. PET and normalized blood tumor markers demonstrated a complete metabolic response inside the liver for 13 months following CAR T-cell therapy, with an abundance of CAR+ cells found inside post-treatment tumor specimens (NCT02850536) (94).

### 4.11 EpCAM

Epithelial cell adhesion molecule (EpCAM) is a type I transmembrane glycoprotein located on the surface of epithelial cells that is implicated in cell adhesion, differentiation, proliferation, and migration (95). EpCAM overexpression has been found in PCa tissues and metastases as compared to benign prostate tissue from PCa patients or prostate tissue from healthy individuals (96, 97). Preclinical investigations in mice employing a cancer xenograft model revealed that intravenous injection of EpCAM-CAR T-cells resulted in considerable tumor control (98–100). However, in one preclinical study, substantial toxicity was reported in BALB/c and C57BL/6 mice receiving EpCAM-CAR T-cells (101). This study’s findings highlight the need of doing adequate preclinical toxicity assessments before introducing novel CAR T-cell therapies into the clinic.

To establish EpCAM as a viable target in human cancer, therapeutic approaches must be improved. Several considerations have been proposed, such as choosing a dose range with few adverse effects and adequate anti-tumor potency, developing a distribution-restricted CAR T-cell that kills tumor cells locally, and enhancing the scFv affinity so that CAR T-cells can only be stimulated by the high density of EpCAM protein on the surface of tumor cells but not on healthy cells (101). EpCAM-CAR T-cells are now being tested in a number of clinical trials for several cancers such as breast cancer, HCC, pancreatic cancer, and GC, though their effectiveness has yet to be demonstrated (NCT02915445 and NCT05028933).

### 4.12 GPC3

Glypican-3 (GPC3) belongs to the glypican family of proteoglycans that are connected to the cell surface through a glycosylphosphatidylinositol (GPI) anchor. GPC3 is essential for cellular differentiation, proliferation, and migration (102). Several studies have shown that GPC3 is an appealing liver cancer-specific target since it is substantially expressed in HCC but not in normal tissues (103). In the Phase I study, the safety and preliminary efficacy of GPC3-CAR T-cells were assessed in 13 Chinese patients with GPC3-positive HCC (NCT02395250). The therapy was well tolerated by all 13 patients who received at least one infusion of GPC3-CAR T-cells. There were no DLTs reported, and there was just one severe adverse event (SAE) of grade-3 fever. This phase I trial demonstrated that GPC3-CAR T-cell is effective and safe for patients with GPC3-positive HCC and that it has robust anticancer potential when combined with lymphodepleting conditioning (NCT02395250) (104). In an open-label, dose-escalation trial, 10 patients were treated with a single infusion of GPC3-CAR T-cells, with the greatest dosage level of 3×10^8^ given to seven patients. The treatment was well tolerated, with no DLTs reported by the nine patients who were followed for at least one month. All subjects experienced a temporary grade-4 reduction in lymphocyte count as a consequence of the lymphodepletion regimen and CRS was observed in eight patients, however, there was no evidence of neurotoxicity (ChiCTR1900028121) (105). Another phase 1 clinical trial used CAR T-cells engineered to express human IL-7 and CCL19 to enhance GPC3-CAR T-cell infiltration and persistence. 30 days after intratumoral delivery of these cells to a patient with advanced HCC, the CAR T-cells with IL-7 and CCL19 integration completely eliminated the tumor (NCT03198546) (106).

### 4.13 B7H3

B7-H3 (CD276), an immune checkpoint molecule, is associated with a poor prognosis and enhances tumor immune evasion and metastatic potential (107, 108).

Recent studies have shown that B7-H3-CAR T-cells have robust antitumor effects in a variety of solid tumor preclinical models, including neuroblastoma, ovarian cancer, PDAC, as well as several pediatric malignancies (109, 110). Several B7-H3-CAR T-cell clinical trials are currently recruiting participants, with the majority of them concentrating on recurrent or refractory CNS malignancies (NCT04185038 and NCT04077866).

## 5 Obstacles to CAR T-cell therapy in solid tumors

Remarkable remission with negligible residual disease in a staggering 61 (81%) of 75 treated patients was the groundbreaking result of a recent clinical trial on patients with acute B-cell lymphoblastic leukemia treated with CD19-specific CAR T-cells (111, 112). Quite contrary to this breakthrough, the after-effect of the administration of first-generation CAR T-cells targeting multiple antigens (carbonic anhydrase IX (CAIX), CD171, folate receptor alpha (FR-α), GD2, HER2, mesothelin, EGFRvIII, or VEGF-R2) (113) failed to yield satisfactory results due to limited activity and recurrent toxicity (114–116). Despite the highly lethal toxicity resulting from TCR-modified T-cells (117), encouraging outcomes arose from the application of a TCR with the cancer-testis antigen, also known as New York esophageal squamous cell carcinoma 1 (NY-ESO-1), as its target (118). Although melanoma, sarcoma, and myeloma all evinced this remarkable result, other responses remained anecdotal (118). Therefore, unlike hematologic malignancies, solid tumors presented obstacles disregarding the deployed approach. A number of barriers hamper the full activation and persistence of T-cells. Firstly, scant infiltration of T-cells can be brought about by the hypoxic, poorly vascularized, and extracellular matrix-rich TME, which also weakens specific recognition owing to tumor antigen loss. In this respect, studies have outlined that tumor cells impair the expression of vasculature-related factors, including overexpression of tumor surface endothelin B receptors in thyroid cancer, which leads to intercellular adhesion molecule-1 (ICAM-1) downregulation and, as a consequence, failure of ICAM-1-redirected CAR T-cells to target advanced thyroid cancer (119). What is more, surface proteins, cytokines, or soluble products of disrupted cell metabolism can have inhibitory roles. The sound evidence of increased lymphocyte infiltration as a determining positive prognosis marker in a range of cancer subtypes (e.g. breast cancer, CRC, ovarian cancer, NSCLC, melanoma, and others) justifies the demand to boost T-cell recruitment in solid malignancies (120, 121). Despite this, post-EGFRvIII-specific CAR T-cell therapy in refractory GB patients disclosed a substantial downregulation of EGFRvIII, which may impede tumor-specific antigen-induced immunogenicity and hence hamper effective CAR T-cell antitumor cytotoxicity in the TME (48). Thus, beyond the challenges of CAR T-cell trafficking to the tumor site, tumor cell heterogeneity and poor immunogenicity are significant obstacles to tackle.

**Figure 1:**
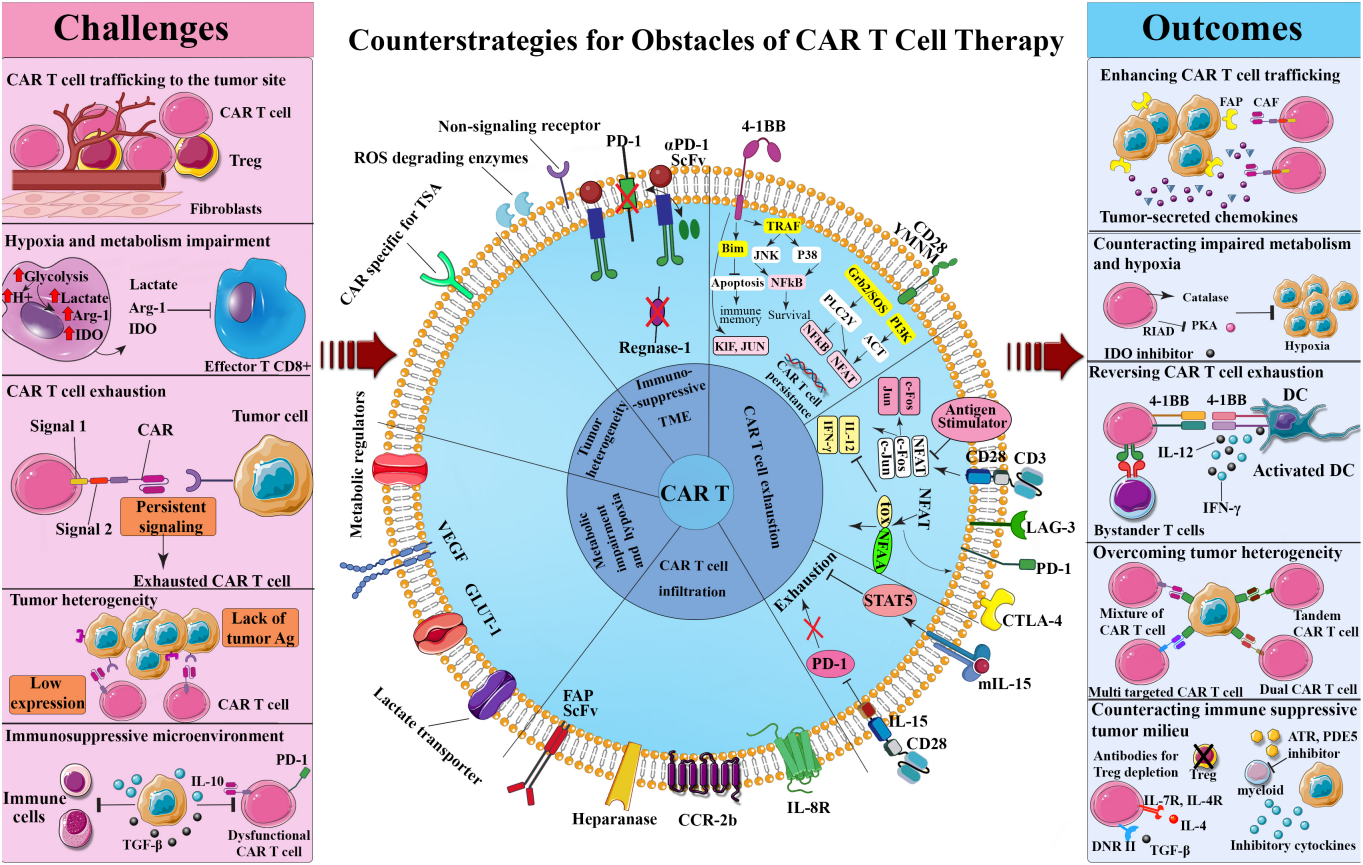
Challenges and counterstrategies of CAR-T-cell therapy. Although CAR T-cell therapy confronts several challenges, such as CAR T-cell trafficking to the tumor site, hypoxia and metabolism impairment, CAR T-cell exhaustion, tumor heterogeneity, and immunosuppressive tumor microenvironment, potential engineering methods and combinational therapies have shown promise in overcoming these obstacles. ATR, All-trans retinoic; CAF, Cancer-associated fibroblast; CAR, Chimeric antigen receptor; CTLA-4, Cytotoxic T lymphocyte-associated antigen 4; DC, Dendritic cell; DNR, Dominant negative receptor; FAP, Fibroblast-activation protein; IDO, Indoleamine 2,3-dioxygenase; IFN-γ, Interferon-gamma; JAK, Janus kinase; LAG-3, Lymphocyte activation gene-3; NFAT, Nuclear factor of activated T-cells; NF-κB, Nuclear factor-κB; PD-1, Programmed death-1; PDE5, Phosphodiesterase 5; PI3K, Phosphoinositide 3-kinases; RIAD, Regulatory subunit I anchoring disruptor; ROS, Reactive oxygen specimens; ScFv, Single chain variable fragment; STAT5, Signal transducer and activator of transcription 5; TGF-β, Transforming growth factor-beta; TOX, Thymocyte selection-associated HMGB; TRAF, TNF receptor associated factor; TSA, Tumor-specific antigen; Treg, Regulatory T cell.

The fact that cancer cells resort to some adaptive strategies to avoid immune detection has faded the significance of the sharpened tumor recognition by T-cells, with MHC-associated antigen presentation being a telling example (122–124). However, there exist heartening outcomes of phase II of the clinical trials of some cancer vaccines, namely Canvaxin and GM-CSF gene modified tumor vaccine (GVAX) in curing melanoma and PCa, respectively which stimulate antigen cross-presentation and variety, culminating in the protective immunity against cancer (125–128). The advantageous clinical effects of Canvaxin and GVAX vaccines could not surpass phase II of the trials and the survival benefits did not appear in the third phase. In the same vein, the results obtained from a multi-peptide vaccine depicted an identical pattern to that of the previously mentioned vaccines once the induction of immune responses commenced; however, low immunogenicity halted further progress in phase III (127). Thus, it can be confirmed that there is a close correlation between the positive impact of vaccination and the induction of a particular immune response; despite this, its efficacy on solid malignancies remains doubtful (129, 130). Outstanding achievement rates of the therapies that single out immune inhibitory checkpoint proteins such as PD-1 further justify the demand for improved T-cell activation and persistence (131, 132). Additionally, the immune suppressor cells in the TME including regulatory T-cells (Tregs), MDSCs, and tumor-associated macrophages (TAMs) elicit a significant inhibitory effect on administered tumor-targeting immune cells while enhancing tumor cells proliferation, angiogenesis, and migration by producing angiogenic, growth, and anti-inflammatory factors (133). The application of engineered T-cells as a plausible treatment for solid tumors is contingent upon cancer’s potential to modify its TME, stir immune cell exclusion, and minimize antigen presentation and lymphocyte activation (134). Nonetheless, over a period of time, CAR T-cells in the TME exhibit phenotypically dysfunctional states such as exhaustion that potentially coincide with inferior clinical outcomes. This challenging phenomenon is triggered by inhibitory Treg-derived cytokines, persistent antigen stimulation, and metabolic stress (135) (**Figure 1**).

## 6 Counterstrategies for obstacles of CAR T-cell therapy

### 6.1 Ameliorating CAR T-cell trafficking to solid tumors

Being unresponsive to immune checkpoint blockade therapy in the biopsy samples is associated with minimal immune infiltration, which serves as a prognostic factor for low rates of survival (136). Unless effector T-cells, such as CAR T-cells, reach the host cells, immunotherapy and consequently tumor control will be futile. Cancer chemokine signaling is a novel technique that elevates the recruitment rate of immune cells at tumor sites, which entails some cytokines capable of modulating the migration and trafficking of a wide range of immune and somatic cells thanks to their chemotactic features (137). The chemokine signals for the proliferation, survival, progression, migration, and drug resistance of cancer cells are emitted either by tumor cells or their stroma (138, 139). What aggravates the TME is the utilization of immune-suppressive cells by such signals (137). However, researchers have directed their attempts to take advantage of the chemokine signaling network to stimulate T-cell recruitment by applying engineering methods to the expression of a cognate chemokine receptor CCR2 (140), CCR2b (140, 141), CCR4 (141, 142), CCR7 (143), CCR8 (144), CXCR1 (145), CXCR2 (146), CXCR4 (146), CXCR6 (147), or CX3CR1 (148) on the surface of CAR T-cells. Today, scientists’ attention has turned to CXCR3 for boosting immune cell recruitment using a CXCR3-ligand-dependent manner subsequent to PD-1 blockade or chemotherapy (149, 150). Sizeable investigations endorse the findings regarding the highly heterogeneous chemokine environment within tumors, highlighting the significance of the identification of the exact candidates for precise infiltration of T-cells into various cancers in a broad spectrum of patients (151). Although directed dispatch of transgenic chemokine receptors to the targeted location is highly sought after, T-cells can be diverted from their route to the designated sites due to the non-tumor specificity of chemokines. It results in the occurrence of novel toxicities and decreased activities, which are mostly considered as the major risks of detecting the primary non-tumor-specific targets (152). When all conditions are stable, there is negligible permeation into non-inflamed or non-tumor tissues, but further toxicities are supposed to stem from disrupted conditions due to injury or autoimmune disorders, necessitating the immediate isolation of patients from clinical tests investigating chemokine-receptor-transduced T-cells (153). The newly developed receptors fiercely rival the endogenously expressed chemokine receptors which T-cells innately use to traffic (154). Unfavorable adverse effects can be averted provided that the detrimental effects of the aberrant chemokine receptors in signaling as well as their homing behavior are taken into account and investigated. On the flip side, when CAR T-cells are absorbed into the recognizable chemokine gradients, chemokine loss or downregulation with modified cancer biology and ineffective immunotherapy are the inevitable outcomes. Efforts should be directed to minimize the heterogeneity of chemokine expression within the patients. Since modified T-cells may fail to detect the inflicted sites for inadequate chemokine expression, a number of intra-tumoral delivery methods are being scrutinized to prompt tumors to escalate the expression rate of the intended chemokine ligand to the desired level. In this way, investigations on animal models of GB, ovarian, and pancreatic cancers have shown that natural or ionizing radiation provokes tumor release of IL-8, which enhances the trafficking of IL-8 receptor-engineered CD-70-CAR T-cells to the tumor site (155, 156). Reaping the benefits of local chemokine delivery and production is a less onerous and more clinically feasible method to streamline the CAR T-cell movement and infiltration; thus, intercompartmental or intratumoral delivery of an adenovirus expressing chemokine (CCL17) (157) or DNA plasmids encoding the favored chemokine ligand (CCL5) is being probed (158, 159). Furthermore, chemo-agent Docetaxel has been shown to upregulate high mobility group box 1 (HMGB1), which stimulates CXCL11 production (160). Additionally, some researchers have employed engineered oncolytic viruses (OVs) to stimulate tumor cell chemokine production, as demonstrated by intravenous delivery of CXCL11-modified oncolytic vaccinia virus to a mouse tumor model, which resulted in an increased intratumoral CXCL11 concentration and thus high CAR T-cell infiltration (161). Similarly, CAR T-cells were combined with an oncolytic adenovirus that was armed with the regulated on activation normal T expressed and secreted chemokine (RANTES) and the cytokine IL-15 in order to overcome the limited T-cell migration and the extremely immunosuppressive TME of solid tumors. In this combinational treatment, oncolytic adenovirus actively augmented caspase pathways in tumor cells exposed to CAR T-cells, and intratumoral secretion of RANTES and IL-15 attracted CAR T-cells and enhanced their local survival, respectively, hence improving the OS of neuroblastoma xenograft mice model (162). In spite of such merits, the acquired results remain mediocre. What aggravates the case is the reluctance of the candidates to undergo the technical complications of reaching the site, not to mention the unyielding resistance of the metastatic site to this treatment (24).

Importantly, CAR T-cell homing competence is substantially reliant on their vigorous adhesion to endothelial cells lineage of vascular beds to prevail hemodynamic shear stress, which is mediated by expression of E-selectin and its cognate ligand sialyl Lewis-X (sLeX) (163). Given the upregulated expression of E-selectin in tumor endothelial beds, reinforcing sLeX expression on CAR T-cells by glycoengineering has been evidenced to facilitate cell trafficking to the tumor site (164). As well, tumor vascular-targeting interventions to remodel tumor vasculature with Bevacizumab (165), disrupt vasculature with combretastatin A-4 phosphate (CA4P) (166), or permeabilize blood-brain barrier (BBB) with intraarterial delivery of NEO100 (167) have been shown to improve CAR T-cell infiltration.

Another strategy for making solid tumors more receptive to CAR T-cells is interfering with T-cell egress. This method relies on heightened α4 integrin signaling that arises from the stabilizing α4 (S988A)-paxillin interaction that develops when α4 (S988A) integrin mutants inhibit protein kinase A (PKA)-mediated phosphorylation. Likewise, through the phenomenon of integrin transregulation, the inhibited PKA-mediated α4 integrin phosphorylation intensifies integrin αLβ2 (LFA-1)-mediated migration; both methods pave the way for T-cells to be extracted from the vasculature into the tissue, rendering the inflamed tissue more open to the adhesion of T-cells in an ICAM-1- and vascular cell adhesion molecule 1 (VCAM-1)-dependent manner (168). *In vitro* studies revealed that inhibiting α4 integrin phosphorylation increased the mass flow of αLβ2-mediated T-cells, while *in vivo* studies found a significant increase in T-cell permeation into ectopically transplanted melanoma tumors and shrinkage of implanted B16 melanoma tumors (169). Target antigens like αvβ3 integrin and vascular endothelial growth factor (VEGF) receptor-2 (VEGFR2), which are profusely expressed in TME and tumor vasculature, respectively are being targeted by the newly engineered CAR T-cells (170, 171).

Currently, researchers are seeking solutions to amplify the receptivity of solid tumors, concealed in dense extracellular matrices, to CAR T-cells. As CAR T-cells infiltrate the tumor site, multiple strategies have been investigated to overcome the physical barriers posed by extracellular matrix (ECM) components and cancer-associated fibroblasts (CAFs). It was found that armoring CAR T-cells with ECM-degrading agents like heparanase (HPSE) enhanced their infiltration and anti-tumor activities (172). Conversely, developing CAR T-cells engineered to target fibroblast-activation protein (FAP), although promotes CAR T-cells trafficking, they induce significant off-tumor toxicities owing to high FAP expression in healthy tissues (173).

Furthermore, preclinical studies have verified the advantageous implications of photothermal therapy in CAR T-cell infiltration *via* antigen spreading, reduction of interstitial fluid pressure, and disruption of tumor tissue structural compaction (174). In this respect, Miller et al. discovered that engineering thermal-specific gene switches results in the potential induction of IL superantagonist or T-cell-redirecting bispecific antibodies that boost anti-tumor responses and limit antigen escape while maintaining key CAR T-cell functions such as proliferation, migration, and cytotoxicity (175).

Alternatively, locoregional delivery of CAR T-cells not only improves their biodistribution to tumor cells with low dosages but also avoids the cytotoxicity induced by systemic delivery routes. In this way, preclinical *in vivo* mouse model studies revealed that intraperitoneally delivered ErbB-targeted CAR T-cells elicited tumor reduction with dose-dependent side effects, whereas when these cells were administered intratumorally or intravenously, there was partial tumor regression with no clinical or histopathological toxicity (176). Likewise, intratumoral, intracranial, and intraventricular delivery of CAR T-cells for the treatment of CNS tumors have been shown to efficiently bypass the BBB and promote anti-tumor activity and tumor regression considerably more effectively and safely than intravenous administration (177, 178). Furthermore, consistent results have been found in phase I clinical trials investigating intrapleural and intrahepatic CAR T-cell delivery methods for the treatment of pleural and liver cancers, respectively (58, 92).

### 6.2 Overcoming hypoxic TME

Tumor cell over-proliferation and chaotic microvasculature potentially confront CAR T-cells with a nutrient-deficient and hypoxic TME, that merits compensative interventions to enhance their anti-tumor activities. Mechanistically, hypoxic condition induces activation of hypoxia-inducible factor (HIF) proteins that enhance tumor cell or MDSCs expression of immune checkpoint receptors PD-1 and V-domain Ig suppressor of T cell activation (VISTA), macrophage immune checkpoint CD47, nonclassical MHC-I molecules, human leukocyte antigen (HLA)-G and HLA-E, as well as Tregs recruitment, resulting in the release of immunosuppressive metabolites (179). Despite this, HIF1 stabilization in T-cells under hypoxic conditions leads to increased production of glycolytic enzymes and glucose transporter 1 (GLUT-1), decreased oxidative phosphorylation, and enhanced VEGF expression (180), all of which contribute to better adaptation of CAR T-cells in hypoxic TME. Several investigations on interventions to augment HIF in CAR T-cells have been undertaken, with the objective of developing CAR T-cells that are selectively sensitive in low-oxygen settings and responsive to antigens upregulated in hypoxic TME, resulting in restricted off-tumor cytotoxicity. In this way, incorporation of the oxygen-sensitive domain of HIF-1α oxygen-dependent degradation (ODD) into the CAR scaffold, termed HIF-CAR (181), HiCAR (182), and HypoxiCAR (183), led to sub-optimal CAR expression in the normoxic setting owing to the hydroxylation and degradation of the CAR as oxygen became available, and on the other hand, high expression of the CAR in the hypoxic condition, enabling CAR T-cells to induce cytotoxic effects on tumor cells specifically. However, HiCAR and HypoxiCAR, in comparison to HIF-CAR, are dual hypoxic sensitive, with a single or consecutive hypoxia response element (HRE) upstream of their promoters triggering CAR expression in hypoxic conditions. Similarly, studies on the hypoxic TME of GB tumors demonstrated an upregulated expression profile of CAIX as a feasible antigen to target by anti-CAIX CAR T-cells with low off-target toxicity (184).

### 6.3 Counteracting metabolic challenges

Metabolic challenges can be classified into two categories: i. dysregulated metabolism of tumor cells due to nutrient-deficient TME, preceded by the production of unfavorable metabolites, impairing effective anti-tumor immunity; and ii. impaired metabolism of CAR T-cells trafficking to TME, significantly impairing their effective functionality (**Table 1**).

**Table 1:** Major challenges and counterstrategies of CAR T-cell therapy in solid tumors.

Challenges	Strategies	Examples	References
**CAR T-cell trafficking to Solid Tumors**	Stimulating tumor cells to produce chemokine and local chemokine delivery	Intravenous delivery of CXCL11-modified oncolytic vaccinia virus	(161)
		Combining CAR T-cells with OV armed with the RANTES and IL15	(185)
		Administering chemotherapy drugs, such as temozolomide, dacarbazine, and cisplatin induced the expression of T-cell-attracting chemokines *in vitro.*	(149)
		Intratumoral delivery of adenovirus expressing chemokine (CCL17) or DNA plasmids encoding the favored chemokine ligand (CCL5)	(157, 159)
	Upregulating expression of chemokine receptors on CAR T-cells that match and respond to tumor-derived chemokines	Engineering CAR T-cells to overexpress CXCR1/CXCR2	(155)
		Modifying integrin αvβ6-CAR T-cells to express CXCR2	(186)
	Engineering CAR T-cells to improve penetration through physical barriers	Armoring CAR T-cells with ECM-degrading agents like heparanase or fibroblast activation protein to overcome the physical barriers posed by cancer-associated fibroblasts (CAFs) and ECM components.	(172, 187)
	Enhancing expression of adhesion molecules on CAR T-cells for strong adhesion to endothelial cells	Reinforcing sLeX expression on CAR T-cells by glycoengineering, given the upregulated expression of E- selectin in tumor endothelial beds	(164)
	Engineering CAR T-cells to enhance penetration through vascular barriers	Administering Combretastatin A-4 phosphate (CA4P), a vascular disrupting agent (VDA)	(166)
		Intra-arterial delivery of NEO100 permeabilized the BBB in a reversible and nontoxic manner	(167)
	Engineering CAR T-cells to enhance penetration through targeting neovasculature	Engineering CAR T-cells to specifically target antigens like VEGF receptor-2 and αvβ3 integrin, which are highly expressed in the tumor microenvironment and tumor vasculature, respectively.	(170, 188)
**Hypoxic tumor microenvironment**	Augmenting HIF in CAR T-cells, which contributes to better adaptation of CAR T-cells in hypoxic TME	Incorporating the oxygen-sensitive domain of HIF-1α ODD into the CAR scaffold, termed HIF-CAR, HiCAR, and HypoxiCAR, enabling CAR T-cells to induce local cytotoxic effects on tumor cells.	(181–183)
**Impaired metabolism of tumor cell**	Engineering CAR T-cells to address hypoxia-adenosinergic immunosuppression	Equipping CAR T-cells with RIAD(RIAD-CAR), which inhibits adenosine-GPCR interaction-induced PKA activation.	(189)
		Administering CAR T-cells in combination with the adenosine A2BR agonist, BAY 60-6583, resulting in synergistic anti-tumor effects.	(190)
	Protecting CAR T-cells from oxidative stress	Equipping CAR T-cells with catalase (CAR-CAT) to improve their antioxidative capacity due to excessive reactive oxygen species (ROS) production.	(191)
	Engineering CAR T-cells to overcome immunosuppressive metabolites such as lactate	Combining CAR T-cells with lactate dehydrogenase (LDH) blockade.	(192)
**Impaired metabolism of CAR T-cell**	Suppressing tumor metabolism (like, reducing glucose uptake) for optimal activity of CAR T-cell	Engineering CAR T-cells to secrete anti-PD-L1 antibodies suppressed Akt-mTOR signaling, resulting in reduced glucose uptake and glycolysis	(193)
		Administering selective GLUT1 inhibitors such as rapamycin (mTOR-signaling inhibitor), metformin, and a ketogenic diet for reducing tumor glucose uptake and thereby improving CAR T-cell metabolism. Employing CRISPR/Cas9 technology to knock out enzymes implicated in tumor metabolism, such as diacylglycerol kinase	(194, 195)
**CAR T-cell exhaustion**	Manipulating the CAR T-cells structure	Grafting CDR into the FR of non-exhaustive scFv, or modification of the spacer between scFv and transmembrane domains, such as designing CARs with just CH3 rather than both CH3 and CH2 in immunoglobulin spacer to restore CAR T-cell exhaustion.	(196, 197)
		Incorporating the 4-1BB as an intracellular domain instead of CD28 to elicit a weak costimulatory signal, leading to lower expression of exhaustion-related genes.	(196)
		Engineering CAR T-cells to express PD-1 dominant-negative receptors or PD-1:CD28 switch receptors, which disrupt PD1 inhibitory signaling and hence render CAR T-cells resistant to PD-L1 overexpressing TME.	(198, 199)
**Tumor heterogeneity**	Designing CAR T-cell to target multiple tumor-associated antigens (TAAs)	Combining separate CAR T-cell products that target specific antigens before administration or by transducing T-cells with numerous CAR constructs.	(200)
	Designing CAR T-cells to co-express and secrete bi-specific T-cell engagers (BiTEs)	Engineering CAR T-cells to secrete BiTEs against EGFR redirected CAR T-cells and attracted untransduced bystander T-cells to eliminate heterogeneous tumors.	(201)
	Administering CARs targeting adapter molecules to connect a variety of soluble antigen-recognition moieties	Combining Avidin-linked CARs in with biotinylated antibodies to manage CAR T-cell function similarly to a safety switch, as well as to target several antigens sequentially or concurrently.	(30, 202)
		Utilizing leucine zipper motifs in the CAR system, to pair CARs (zipCAR) with free scFvs (zipFv), allowing for simultaneous targeting of various antigens.	(203)
**Immunosuppressive microenvironment**	Interfering with immunosuppressive cytokines and inhibitory signaling pathways	Designing CAR T-cells to express IL-4 receptors alone or in tandem with receptors for IL-7 and TGF-β to target suppressive cytokines in the TME	(74)
	Engineering CAR T-cells to release immunostimulatory cytokines or CARs resistant to immunosuppressive factors	Developing CARs to produce immunostimulatory signals has depended on IL-12 production and redirecting immunosuppressive cytokines (including IL-4), leading to enhanced survival, proliferation, and anticancer activity.	(204, 205)
	Combination of CAR T-cells and immune checkpoint blockade	Designing CAR T-cells capable of producing anti-PD-L1(anti-CAIX CAR T-cell)	(193)
	Depleting or redirecting immune suppressor cells in the TME	Several phase I clinical trials have applied concurrent lymphodepletion chemotherapy (like Fludarabine, and cyclophosphamide) with CAR T-cell therapy in solid tumors.	(206, 207)
		Combining CAR T-cells with agents that suppress MDSCs, such as all-trans retinoic acid.	(208, 209)

#### 6.3.1 Impaired tumor metabolism

The hypoxic TME contributes to increased adenosine triphosphate (ATP) breakdown and suppression of adenosine kinase, resulting in adenosine accumulation that interacts with its specific receptors A2AR and A2BR, inhibiting T-cells’ anti-tumor immune responses (210). Several studies have addressed hypoxia-adenosinergic immunosuppression by arming CAR T-cells with regulatory subunit I anchoring disruptor (RIAD), known as RIAD-CAR, which inhibits adenosine-G-protein-coupled receptor (GPCR) interaction-induced PKA activation (189), or by administering CAR T-cells in combination with the adenosine A2BR agonist, BAY 60-6583 (190), resulting in synergistic anti-tumor effects. As well, excessive production of reactive oxygen specimen (ROS) as a consequence of alterations in metabolic pathways (e.g., glucose deprivation) has prompted researchers to equip CAR T-cells with catalase (CAR-CAT) to enhance their antioxidative capacity (191, 211). Furthermore, excessive aerobic glycolysis of tumor cells increases the synthesis of lactate, an immunosuppressive metabolite in TME (212). To overcome this metabolic barrier, researchers investigated lactate dehydrogenase (LDH) blockade in conjunction with CAR T-cell immunotherapy and discovered a substantial improvement in CAR T-cell therapeutic efficacy in a mouse model of PCa (192).

#### 6.3.2 Impaired CAR T-cell metabolism

Given that tumor cells overconsume nutrients in the TME, there is competition for energy resources between tumor cells and CAR T-cells, which emphasizes the significance of strategies to suppress tumor metabolism for optimal CAR T-cell activity (213). For instance, PD-L1 overexpression by tumor cells mediates Akt-mTOR activation, resulting in increased glucose uptake and glycolysis. It suggests that PD-1/PD-L1 axis blockade might be a potential approach to optimize CAR T-cell metabolism and hence expand their cytotoxicity (193, 214). Additionally, selective GLUT-1 inhibitors such as rapamycin (mTOR-signaling inhibitor), metformin, and a ketogenic diet may reduce tumor glucose uptake and thereby improve CAR T-cell metabolism in the TME (194). Similarly, CRISPR/Cas9 technology has been applied to knock out enzymes implicated in tumor metabolism, such as diacylglycerol kinase, with encouraging outcomes in ameliorating CAR T-cells immunometabolism (195).

From another perspective, the subset fate of CAR T-cells is greatly influenced by their metabolic profile, as CAR T-cells with enhanced glycolytic metabolism tend to differentiate into the effector subset with high invasive potential and low persistence, and conversely, CAR T-cells with the metabolic shift towards oxidative phosphorylation (OXPHOS) tend to differentiate into the memory subset with low migratory and killing capacity but greater persistence (215). In light of this, restricting glycolytic metabolism throughout the manufacturing procedures and promoting it at the tumor site would be the essential strategy to maximize the therapeutic efficacy of CAR T-cells. During CAR T-cell manufacturing procedures, direct inhibition of glycolysis by interfering with the PI3K/AKT/mTOR signaling pathways using the selective phosphoinositide 3-kinase (PI3K) inhibitor Idelalisib (CAL-101) (216) or the selective PI3K (p110) isoform inhibitor GSK2636771 (217), as well as indirect inhibition of glycolysis by optimizing CAR T-cell media through the addition of IL-15 (218) or IL-21 (219), can potentially lead to low T-cell differentiation and memory T-cell development. In addition, L-arginine supplementation of CAR T-cell culture media or equipping CAR T-cells with arginine resynthesizing enzymes was found to shift CAR T-cell metabolism from glycolysis toward OXPHOS (220, 221). Alternatively, transient glucose restriction in the culture media of CAR T-cells led to increased persistence in the TME and enhanced production of tumor-targeting molecules IFN-γ and granzyme B (222). Besides that, the costimulatory domain included in the CAR structure is a regulator of the metabolic pathways in CAR T-cells, as CD28, OX40, and inducible co-stimulator (ICOS) stimulate glucose uptake and glycolysis, whereas 4-1BB exploit the OXPHOS pathway (215, 223). As a consequence, 4-1BB CAR T-cells have a superior metabolic profile and differentiate in central memory T-cells, which proliferate slowly but persistently. On the flip side, glycolysis in CAR T-cells after administration can be potentially provoked by inhibition of TGF-β in the TME using CRISPR/Cas9 technology (224) or engineering CAR T-cells to express dominant negative TGF-β receptor (225). Similarly, AKT downregulation in the TME suppresses glycolysis, indicating that AKT upregulation in the TME might promote glycolysis. In this way, researchers have investigated CAR T-cells that overexpress AKT and demonstrated promising anti-tumor activity (226). Nonetheless, these techniques may augment terminal differentiation in CAR T-cells while reducing their persistence, highlighting the significance of large-scale research to optimize compensatory measures.

### 6.4 Reversing CAR T-cell exhaustion

Comparative studies of the superior clinical outcomes of CD19-specific CAR T-cells in hematologic malignancies versus solid tumors have shown CAR structural differences that may be implicated in the exhausted phenotype of CAR T-cells in solid tumors. In this context, CD19 CAR designs have been established to incorporate a particular scFv that, unlike other CARs, does not exhibit self-aggregation, which is characterized by CAR T-cell activation independent of antigen exposure. These findings point to the significance of inhibitory strategies for tonic signaling in CAR T-cells (227). The main strategies encompass manipulation of the scFv structure or transient inhibition of CAR expression. In this way, complementarity-determining region (CDR) grafting into the human framework (FR) of non-exhaustive scFv, such as CD19 scFv (196), or modification of the spacer between scFv and transmembrane domains, such as designing CARs with just CH3 rather than both CH3 and CH2 in immunoglobulin spacer (197), have been shown to restore CAR T-cell exhaustion. In addition, incorporation of the 4-1BB as an intracellular domain (GD2.BBz CAR) instead of CD28 (GD2.28z CAR) has been shown to elicit a weak costimulatory signal while still enabling T-cells activation upon antigen exposure (228), resulting in lower expression of exhaustion-related genes (196), higher expression of memory-related transcription factors (196), increased oxidative metabolism (223), and long-term survival of CAR T-cells (1). Furthermore, preclinical investigations have demonstrated that introducing a destabilizing domain (DD) into the CAR design allows for tonic signaling modulation by CAR degradation or otherwise neutralization of DD functionality through stabilizing reagent delivery (229, 230). Alternatively, some strategies that target CAR signaling rather than CAR constructs, such as dasatinib-mediated inhibition of proximal CAR signaling have shown promise in reversing CAR T-cell exhaustion (231).

On the other hand, exhausted CAR T-cells in the TME are characterized by the upregulated expression profile of inhibitory receptor PD-1, transcription factors thymocyte selection-associated HMGB (TOX) and NR4A, and CBL-B as well as the release of cytokines like TGF-β that contribute to immune exhaustion. These findings merit considerations in developing CAR design or adjunctive combinational therapies to target T-cell intrinsic pathways (232). In this respect, several studies have indicated combinational therapy using CAR T-cells and PD-1 blockade *via* anti-PD-1 antibodies (198), short hairpin RNA (shRNA) or CRISPR-Cas9 (59) mediated silencing of *PDCD1*, as well as engineering CAR T-cells to express PD-1 scFv (233), offer favorable effects on CAR T-cells exhaustion. Other researchers have designed CAR T-cells to express PD-1 dominant-negative receptors (198) or PD-1:CD28 switch receptors (199, 234, 235), which disrupt PD-1 inhibitory signaling and hence render CAR T-cells resistant to PD-L1 overexpressing TME. In a somewhat different strategy, an oncolytic adenovirus was combined with a helper-dependent adenovirus expressing a PD-L1 blocking mini-antibody to restore T-cell dysfunction by inhibiting PD-1: PD-L1 interaction (236). As well, experiments on solid tumor-bearing mice models demonstrated that triple knockdown of transcription factors involved in the expression of inhibitory receptors such as NR4A1, NR4A2, and NR4A3 enhance anti-tumor activity and survival of CAR T-cells (237). Likewise, the TGF-β signaling cascade induces suppression of T-cell expansion, effector function, and migration through upregulation of the inhibitory receptors PD-1 and T-cell immunoglobulin and mucin domain 3 (TIM-3) (238), which have been shown to implicate in various solid tumors development and progression (239). This has prompted researchers to engineer CAR T-cells to co-express a bispecific protein of anti-PD-1 fused with a TGF-β trap to concurrently hinder the PD-1/PD-L1 axis and the TGF-β signaling cascade (233). TGF-β CAR T-cells were also designed to bind TGF-β in the TME, sequester it, and release costimulatory cytokines to counteract TGF-β-mediated immunosuppression (240, 241). In addition to the TGF-β ligand, other components of the TGF-β signaling cascade including TGF-β receptor II (TGFBR2) are a potential target to boost CAR T-cell cytotoxicity in solid tumors. In phase I clinical trial, Narayan et al. armored PSMA-targeting CAR T-cells with a dominant-negative TGF-β receptor and observed a substantial improvement in clonal CAR T-cell expansion and tumor regression (242). TGFβR-KO CAR-EGFR T-cells targeting TGFβR2 are also being investigated to enhance the anti-tumor activity of CAR T-cell therapy in EGFR-overexpressing solid tumors, though results have yet to be published (NCT04976218). Furthermore, CRISPR-Cas9-directed deletion of CBL-B, a negative regulator of immune activation, was shown to restore exhaustion and enhance IFN-γ and TNF-α levels and cytotoxicity in CAR T-cells (243).

### 6.5 Overcoming tumor heterogeneity

Multiple novel engineering approaches have been explored to improve CAR T-cell efficacy by overcoming the antigen heterogeneity of solid tumors. Some of the examples include CAR T-cell engineering to target multiple TAAs, the use of CAR T-cells designed to co-express and secrete BiTEs, and the application of CARs targeting adapter molecules that can bind to a variety of soluble antigen-recognition moieties to facilitate the recognition of several antigens with a single CAR simultaneously (244).

BiTEs are generally composed of two scFvs, one specific to CD3 and the other to a TAA, joined by a flexible linker, and can therefore physically attach a T-cell to a tumor cell (244). BiTE-secreting CAR T-cells have been shown in preclinical models of solid tumors to be successful in overcoming antigen heterogeneity and bypassing antigen escape (36). For example, in this context, a bicistronic design was developed to drive the expression of an EGFRvIII-specific CAR and an EGFR-targeting BiTE. CART.BiTE cells release EGFR-specific BiTEs, which redirect CAR T-cells and attract untransduced bystander T-cells to attack wild-type EGFR. EGFRvIII-specific CAR T-cells were found to be unable to effectively eradicate tumors with heterogeneous EGFRvIII expression, resulting in the outgrowth of EGFRvIII-negative and EGFR-positive GB. While, CART.BiTE cells were shown to eradicate heterogeneous tumors in GB mice models (201). In another study, CART cells that target the FR-α were demonstrated to effectively infiltrate xenograft tumors but were unable to elicit complete immune responses, most likely because of the presence of antigen-negative cancer cells. Therefore, in the absence of FR-α, BiTEs released by oncolytic adenovirus (OAd)-infected cells redirected CAR T-cells toward EGFR, addressing tumor heterogeneity (245). Furthermore, it was shown that, as compared to CAR T-cells, BiTE T-cells exhibit substantial activation, cytotoxicity, and cytokine secretion in response to target-positive gliomas (246).

Several approaches have been designed to construct universal CARs that employ adaptor elements as ligands to allow for the targeting of various antigens with a single CAR T-cell subset. For instance, avidin-linked CARs (also known as biotin-binding immune receptors) in conjunction with biotinylated antibodies can be utilized to control CAR T-cell function similar to a safety switch, as well as to target several antigens sequentially or concurrently (30, 202).

Several researchers have investigated so-called “logical gating” techniques, in which T-cells are activated by either the simultaneous expression of two antigens (A + B) or the expression of antigen A but not antigen B (A-B). These techniques may enable the targeting of antigens produced by both tumor cells and normal cells, provided that the antigen combination chosen is tumor-specific. In this way, Kloss et al. pioneered the “A+B” strategy, employing independent CARs to activate CD3z and the co-stimulatory signal (28). Subsequently, leucine zipper motifs were utilized in the SUPRA (split, universal, and programmable) CAR system, to pair CARs (zipCAR) with free scFvs (zipFv), allowing for simultaneous targeting of various antigens as well as the addition of several antigen logic gates and mitigation of CAR T-cell activation (203). Furthermore, CARs incorporating Fcγ receptors (FcγRs) as the antigen-binding domain enable therapeutic TAA-binding antibodies to target multiple antigens with a single CAR molecule (247). Similarly, CARs with scFvs that identify a fluorescein isothiocyanate fluorophore coupled to TAA-binding molecules were used to target multiple antigens at the same time (23, 248–250).

Another study found that the production of BiTEs targeting a second tumor antigen by an oncolytic adenovirus might overcome antigen heterogeneity (251). The combination of a CAR T-cell with the OV-BiTE triggered activation of T-cells in the lack of the CAR T-cell targeted antigen or CAR expression (i.e., non-transduced T-cell population) (252). Also, OVs can trigger immunogenic cell death (ICD) through the release of HMGB1 and heat shock protein (HSP)70/90, calreticulin exposure, and ATP secretion, upon penetration into tumor cells, enabling the immune system to act more effectively against virus-infected tumor cells to circumvent antigen heterogeneity (253). Membrane-integrated T-cell engagers (MiTEs), like BiTEs, direct T-cells to the surface of tumor cells *via* a CD3 scFv, resulting in tumor cell lysis. However, since MiTEs persist on tumor cell membranes, only tumor cells infected with an OV equipped with MiTEs would be targeted for lysis. While this confines T-cell activation to OV-infected cells, it may reduce on-target and off-target effects compared to BiTEs since activation is dependent on viral tropism rather than the expression of a specific surface marker (254).

### 6.6 Counteracting immunosuppressive TME

Several investigations have been performed with the objective of resisting TME-induced immunosuppression in CAR T-cell therapy by interfering with immunosuppressive cytokines and inhibitory signaling pathways, increasing pro-inflammatory cytokine release, and depleting or redirecting immune suppressor cells in the TME (255). The overproduction of anti-inflammatory cytokines in the TME has been addressed by modifying CAR T-cells to express their corresponding receptors. In this regard, similar to the strategies for TGF-β in the TME outlined above, CAR T-cells have been designed to express IL-4 receptors alone or in tandem with receptors for IL-7 and TGF-β to target immunosuppressive cytokines in the TME (74). Alternately, engineering CAR T-cells to release pro-inflammatory cytokines IL-12, IL-15, IL-18, or IL-21 (204, 256), or integrating CD28 costimulatory domain into the CAR structure to stimulate the release of IFN-γ, GM-CSF, and TNF have shown promise in the anti-tumor activities of CAR T-cells in the immunosuppressive TME (257, 258). Similarly, experiments on mouse models of HCC revealed that co-administration of CAR T-cells with Sorafenib, a tyrosine kinase inhibitor, increases TAM IL-12 production, resulting in superior CAR T-cell activity (258). Another strategy involved combining mesothelin-redirected CAR T-cells with an oncolytic adenovirus expressing TNF-α and IL-2 (OAd-TNF-α-IL-2). In PDAC xenograft immunodeficient mice, Ad-TNF-α-IL-2 boosted both CAR T-cell and host T-cell infiltration to the tumor site and changed host tumor immunological condition with M1 polarization of macrophages and augmented dendritic cell maturation (259). Furthermore, multiple studies have been undertaken to explore strategies for targeting inhibitory signaling pathways induced by interactions between immune checkpoint molecules expressed by tumor cells and their corresponding receptors on immune cells. In this regard, PD-1/PD-L1 axis blockade has been achieved by either redirecting CAR T-cells to release mAbs targeting the PD-1/PD-L1 or knocking out the PD-1 expression on the CAR T-cells (260). For instance, in mouse models of solid tumors, researchers developed CAR T-cells capable of producing anti-PD-L1 (anti-CAIX CAR T-cell) (193) or anti-PD-1 mAbs (CAR.PD-1 T-cell) (261) with superior anti-tumor immunity. Meanwhile, combination therapy with mAbs against the PD-1/PD-L1 axis has been evidenced to have drawbacks such as short-term immunity, reliance on repetitive treatment, and significant autoimmune toxicity (262). As an alternative, CRISPR/Cas9 gene editing of PD-1 expression in CAR T-cells compensates for these drawbacks and allows CAR T-cells to persist longer with a superior safety profile (263). Despite this, some investigations have indicated non-engineering approaches, such as IFN-γ treatment, that, while increasing PD-L1 expression by solid tumors, would also pave the way for bypassing PD-L1/PD-1 by enhancing ICAM-1 expression on tumor cells (264). However, the effects of IFN-γ treatment were not only confined to tumor cells; priming HER2-CAR T-cells were also shown to upregulate the expression of survivin, an anti-apoptotic protein linked to the pathogenesis of multiple malignancies and autoimmune diseases (265, 266), which improved their persistence and anti-tumor cytotoxicity (264). Aside from the PD-1/PD-L1 axis, several studies have been conducted on additional immune checkpoints. In this context, CD-70 CAR T-cells (267) and B7H3-CAR T-cells (268) have been investigated for GB treatment, with encouraging tumor regression results. Similarly, CAR T-cells that target CD-47, a macrophage immune checkpoint, have demonstrated significant anti-tumor immunity in the treatment of CD-47-positive pancreatic, ovarian, and melanoma tumors (269). Several clinical trials are also currently underway to evaluate the anti-tumor efficacy of CAR T-cells producing antibodies against CTLA-4 and PD-1 in MUC1^+^, EGFR^+^, and mesothelin^+^ solid tumors (NCT03179007, NCT03182816, and NCT03182803).

Another strategy for addressing the immunosuppressive TME is to deplete immune suppressor cells in the TME. In this way, several phase I clinical trials have applied concurrent lymphodepleting chemotherapy with CAR T-cell therapy in solid tumors. The most common therapeutic regimens for these conditions are either alone or combinations of Fludarabine, Nab-Paclitaxel, and cyclophosphamide, which has shown promising anti-tumor efficacy while exhibiting no events of grade-3 neurotoxicity or CRS (104, 206, 207, 270–272), except for one case (6%) of grade-3 CRS in a study assessing the combination of EGFR-specific CAR T-cell with Nab-Paclitaxel and cyclophosphamide in 17 patients with stage 4 biliary tract cancers (273). Besides, studies have shown that depleting MDSCs using Gemtuzumab ozogamicin immunotoxin (274) or sunitinib (275) has a synergistic effect on CAR T-cell immunity. Furthermore, observations of pre-clinical models of pediatric sarcoma xenografts are indicative of the fact that the efficacy of CAR T-cell therapy is largely impacted by targeting the infiltrating MDSC population in the TME. Anti-tumor lymphocyte function can be restored as immunosuppressive immature myeloid cells set out differentiating as confirmed by the findings of pre-clinical studies of all-trans retinoic acid (ATRA) therapy (276). Similarly, when GD2-CAR T-cell therapy was combined with ATRA therapy in osteosarcoma xenografts, both the frequency and function of tumor-infiltrating MDSCs plunged, bringing about a general enhancement in survival as opposed to the mice treated with GD2-CAR T-cells alone (208).

## 7 CAR T-cell toxicities

### 7.1 Clinical epidemiology

As the use of adoptive T-cell therapies increases, it is crucial to recognize the particular toxicities of these therapies, which are distinct from those observed with traditional chemotherapies, mAbs, and small-molecule targeted therapies. CRS and immune effector cell-associated neurotoxicity syndrome, which is now more commonly known as ICANS, are the two most frequently observed toxicities associated with CAR-T cell therapy (277, 278). The stimulation of T-cells and subsequent production of cytokines, as well as the attraction and stimulation of other immune cells result in CRS, which is a constellation of inflammatory symptoms (279). These cytokines, which include IL-6, IL-10, IL-2, and IFN-γ, may be released either directly by the CAR T-cells or indirectly by other cells, including monocytes and macrophages, in response to cytokines secreted by the CAR T-cells. The major constitutional symptom of CRS is fever, although other symptoms can involve any organ system and can vary widely, including complications in the digestive, cardiovascular, urinary, respiratory, and nervous systems (280).

According to a meta-analysis of 2592 patients from 84 eligible research, the mortality rate of CRS was less than 1%. Patients with hematologic cancers had a greater CRS rate (grade-3) than those with solid tumors (19%, 95% CI: 8-31%). More than half of the patients were shown to have mild to severe symptoms, which resolved within a few days, as well as nearly 30% of participants eventually experienced severe CRS (grade 3 or above) and required tocilizumab or dexamethasone treatment (281). Similarly, another meta-analysis of 997 patients from 52 studies revealed that the CRS rate was much greater in patients with hematologic malignancies than in those with solid tumors (282). According to meta-analysis research that used subgroup analysis of anti-CD19 CAR T trials, a greater CAR T-cell infusion dosage was a significant factor in raising the incidences of CRS and neurological symptoms. Additionally, children and young patients were at risk for CRS while adults had a higher risk for neurological symptoms. The toxicity associated with CAR T-cell therapy was also impacted by racial or regional disparities. A lower CRS rate and higher neurological symptom incidences were observed more in the USA than in China. Likewise, the origin of the allogeneic T-cells used in CAR T-cell synthesis was a significant factor in the development of CRS, with patients receiving CAR T-cells of allogenous origin exhibiting a higher incidence of CRS. Furthermore, compared to CD28 or 4-1BB co-stimulation, the CRS rate was dramatically reduced by the third generation of CAR T-cell utilizing CD28 and 4-1BB co-stimulations (281). Due to a lower activation threshold, the CD28 costimulatory domain appears to give a more antigen-specific activation of T-cells, perhaps resulting in higher frequencies of CRS. In addition, compared to lentivirus or retrovirus, the gamma retrovirus vector increased ≥ grade-3 CRS incidence (281).

The second most frequent adverse effect of CAR T-cell therapy is neurotoxicity. Neurotoxicity frequently causes seizures, severe encephalopathy, and even death (280). Varied CAR constructs induce different neurotoxicities in terms of frequency and severity. Younger individuals, those who have had a lot of pre-treatments, and patients with pre-existing neurological problems may be more likely to have neurotoxicity (283, 284).

Neurotoxicity’s precise underlying mechanisms are not fully known. One theory is that neurotoxicity is a consequence of the passive diffusion of cytokines into the brain in the context of a permeable BBB as most neurotoxicity is preceded by CRS. On the other hand, a recent study showed that individuals with severe neurotoxicity had significantly higher levels of IL-6, IL-8, IFN-γ-induced protein 10 (IP10), and MCP-1 in their cerebrospinal fluid (CSF) relative to their serum. Additionally, there were higher concentrations of endogenous excitatory neurotransmitters in the CSF of those individuals, including glutamate and quinolinic acid (285).

### 7.2 Management

With precise and timely diagnosis and patient management, adverse outcomes are probably avoidable. Overlapping conditions make it difficult to manage these patients. Antipyretics and fluids are used as symptomatic treatments for low-grade CRS. Given the possibility of vascular leakage and subsequent pulmonary edema, caution should be given when replacing large amounts of intravenous fluid (286). The mainstay of CRS therapy has been the selective blockade of IL-6 signaling by tocilizumab (IL-6 receptor antagonist) or siltuximab (chimeric anti-IL-6 mAb), which results in rapid resolution of CRS symptoms, usually within a few hours. In June 2018, the European Medicines Agency (EMA) approved tocilizumab for the treatment of severe CRS (278, 287). According to clinical studies, tocilizumab is effective in treating CRS caused by CAR-T cell therapy for B-cell acute lymphoblastic leukemia (288).

It is worth noting that tocilizumab does not appear to impair CAR T-cell efficacy in terms of overall response rates, complete response rates, or response duration (289). Tocilizumab use, however, may increase IL-6 level and raise the possibility of severe neurotoxicity. Also, tocilizumab may intensify long-term immunosuppression. Additionally, repeated tocilizumab treatment was associated with a greater prevalence of lower intestine perforations in rheumatic diseases, which is presumably not the case in an acute context (290).

Glucocorticoids have been proven effective in reducing CRS, owing to their capacity to inhibit inflammatory responses. Nevertheless, current studies indicate they may reduce the effectiveness of the CAR T cells (291). Given that IL-1 appears to play a significant role in the development of neurotoxicity and that IL-1 inhibitor (anakinra) has been demonstrated to be efficacious in a mouse models, some researchers also advocate the use of anakinra (292). In refractory cases, IL-1R inhibitors (293), TNF-α blockers, arctigenin (ATG), and T-cell depleting alemtuzumab (292), or cyclophosphamide (111), GM-CSF inhibition (294), and ibrutinib (295), might be helpful, while data is limited to case reports. As well, a recent case report of a 10-year-old B-cell acute lymphoblastic leukemia (B-ALL) child with severe CRS with concomitant neurotoxicity who was resistant to tocilizumab, high-dose steroids, and immunoglobulins demonstrated that hemofiltration might quickly reduce inflammatory markers and alleviate symptoms (296).

Similar to how CRS is managed, patients with mild (grade ≤1) neurotoxicity should be continuously evaluated and given supportive treatment as necessary. Transfer to an intensive care unit or intermediate care unit should be considered for individuals who experience more severe neurotoxicity (grade≥2) (286). The identification of additional causes of neurological symptoms, such as cerebral hemorrhage, CNS involvement of the underlying malignancy, stroke, infection, and others, requires consultation with a neurologist or neurointensivist as well as a lumbar puncture and radiological imaging (297).

Depending on the grade of neurotoxicity, corticosteroids are administered in increasing doses as part of the therapy. There is presently inadequate high-quality clinical evidence to make recommendations for therapy for patients who do not respond to high doses of steroids. The time to resolution of neurotoxicity (range, 4-21 days) was longer than the time to resolution of CRS (range, 0-3 days) after tocilizumab and/or steroid therapy, reflecting that neurotoxicity is less responsive to these interventions than CRS (298). As a suggested therapy, 10 mg of dexamethasone should be taken every six hours until the symptoms go away. While, high doses of methylprednisolone (such as 1000 mg/24h) should be administered in cases with grade-4 toxicity (286).

Given that some individuals had abnormal electroencephalogram (EEG) findings without complaining of any seizure symptoms, a preventative course of antiepileptic medication can also be taken into consideration (286).

## 8 Future perspectives

Synthetic biology and cell engineering have virtually no limits now, offering a platform for the development of novel therapies. Intriguing strategies are currently being developed to improve the efficacy of CAR T-cell therapy. These potential engineering techniques for optimizing CAR T-cell biology will lead to more widespread use of this technology in anti-cancer therapy. However, increasing the complexity of CAR designs and editing the genes on T-cells may increase the risks associated with CAR T-cell therapy. For instance, the use of gene-editing tools and viral transduction both carry the risk of off-target disruption of genes (299). Indeed, a well-anticipated theoretical danger of any gene therapy is the transformation of T-cells into malignant clones due to insertional mutagenesis involving either activation of endogenous proto-oncogenes by viral promoters or loss of tumor-suppressor genes (244).

The CAR gene was specifically inserted into the TET2 locus, causing a clonal increase of T-cells (300). This clonal T-cell population later spontaneously contracted; however, this occurrence emphasizes the danger of using genetically-modified cells to treat patients. A first-in-human trial has been started to assess a transgenic TCR T-cell product directed by NY-ESO-1 that has undergone multiple CRISPR-Cas9 gene edits to eliminate endogenous TCR and PD-1 (301). The numerous unmet requirements in CAR T-cell therapy will be addressed thanks to developments in CRISPR-Cas9-based genome editing (302).

It will be possible to better identify the long-term risks associated with the developing area of gene-editing in medicine and may make it easier to discover remedies for these issues if ongoing monitoring of gene-editing-related complications is done in trials of CAR T-cell products. The price of generating CAR T-cells, which is currently costly, may increase due to novel engineering techniques. However, there are ways to lower manufacturing costs, such as the use of non-viral vectors, which could assist to increase affordability. The expense of CAR T-cell manufacture, particularly the significant cost and time involved with the development of clinical-grade retroviruses is just one of several factors that are reflected in the ultimate cost of these treatments (303).

Because the clinical trial cycle for CAR T-cells is shorter than for other drugs, the only thing we can say with certainty is that within the next 5-10 years, several CARs will be approved for various diseases.

## Author contributions

All authors contributed to the conception and the main idea of the work. AD, LM, and AS drafted the main text, figures, and tables. AM edited the text and designed the table and figure. AA-M revised and edited the text. LA-M and BB supervised the work and provided the comments and additional scientific information. All authors contributed to the article and approved the submitted version.
